# Traditional medicine and natural product based therapeutics in gingivitis management and microbial interactions

**DOI:** 10.3389/frabi.2026.1764314

**Published:** 2026-04-06

**Authors:** Pranita Rath, Sudipta Kumar Patra, Manisha Dash, Sandeep Kumar Behera, Shibani Mohapatra, Kabir Suman Dash, Alok Kumar Panda

**Affiliations:** 1 Kalinga Institute of Dental Sciences, Kalinga Institute of Industrial Technology Deemed to be University, Bhubaneswar, Odisha, India; 2 Department of Orthopedics, Kalinga Institute of Medical Sciences, Bhubaneswar, Odisha, India; 3 Environmental Science Laboratory, School of Applied Sciences, Kalinga Institute of Industrial Technology, Deemed to be University, Bhubaneswar, Odisha, India; 4 Center for Biotechnology, Siksha ‘O’ Anusandhan Deemed to be University, Bhubaneswar, Odisha, India; 5 Centre for Water Research and Climate Change, Kalinga Institute of Industrial Technology, Deemed to be University, Bhubaneswar, Odisha, India

**Keywords:** gingivitis, medicinal plants, microbial interactions, phytochemicals, traditional medicine

## Abstract

Gingivitis is a reversible inflammatory condition of gingival tissues and is majorly driven by the formation and accumulation of microbial biofilm. Untreated gingivitis often leads to periodontitis and ultimate tooth loss. The management of gingivitis through conventional methods relies mainly on the use of chemical antiseptics and antibiotics and the removal of plaque. However, the long-term use of the chemicals and antibiotics may alter the microflora and may also lead to antimicrobial resistance. This has led to increased interest in the usage of natural products-based herbal and traditional medicine as safer alternative medicine. This review provides a comprehensive overview of the pathogenesis of gingivitis with an emphasis on the microbial interaction and conversion of the microflora as the disease progresses. It evaluates the potential of major medicinal plants and their bioactive components used to cure the disease gingivitis. The antimicrobial, anti-inflammatory, antioxidant, and tissue-healing mechanisms of these medicinal plants are discussed alongside evidence from clinical trials. The review further highlights the limitations, such as the lack of standardized formulations and dosage variability differences among the various traditional and herbal medicinal practitioners, which prevents universal adoption of traditional medicine for treating gingivitis. In addition, advanced technologies such as the use of artificial intelligence for developing odontonutraceuticals, and next-generation polyherbal have also been explored. This review established the traditional and herbal medicine as an effective strategy for treating and managing gingivitis.

## 1 Introduction

Gingivitis represents the most prevalent plaque-induced inflammatory oral condition affecting millions worldwide. It is characterized by reversible inflammation of the gingival tissue in response to bacterial biofilm accumulation. According to the World Health Organization (WHO), this is the most common oral disease worldwide, affecting approximately 20% of middle-aged adults and often leading to tooth loss (Hamid et al., 2021). Plaque-induced gingivitis is the most common form of periodontal disease and is one of the most prevalent human inflammatory diseases, which is clinically characterized by gingival erythema, edema and bleeding. These manifestations typically appear 12 to 21 days after uninterrupted dental plaque accumulation and critically occur without the loss of periodontal attachment. The mouth harbors a natural microflora, which generally consists of beneficial microbes, but in the absence of oral hygiene, harmful microbes can accumulate inside the mouth. This leads to a shift in the microbiota, which can lead to several oral health problems including gingivitis. Gingivitis typically presents as an asymptomatic condition with frequent spontaneous gingival hemorrhage, resulting in poor patient awareness and limited recognition of the disease (Fan et al., 2021; Alfahmi et al., 2024). The common symptoms of gingivitis include puffy and swollen gums.

**Figure 1:**
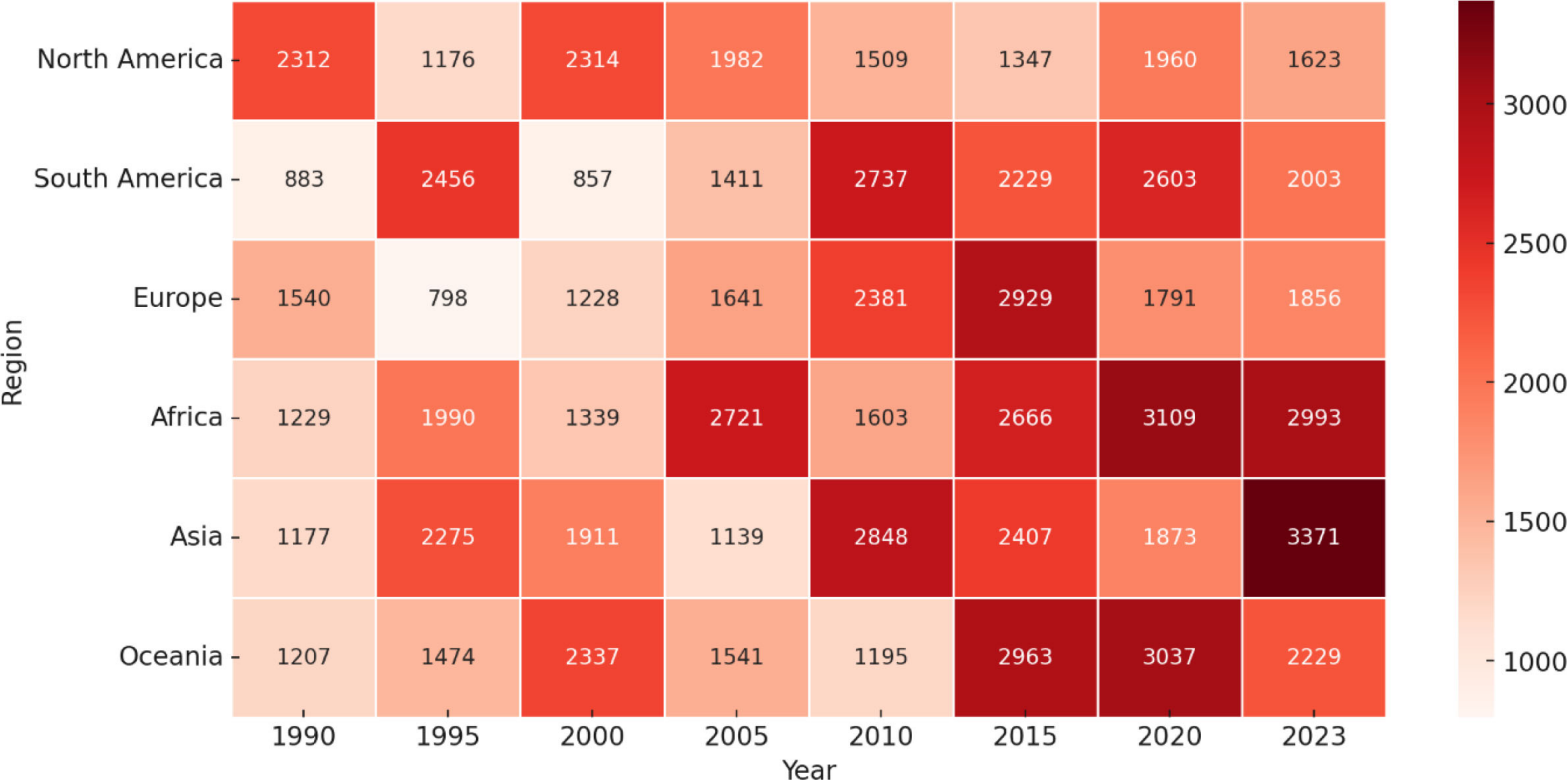
Figure displaying the progression of a variable (e.g., periodontal disease prevalence or healthcare burden) across different regions from 1990 to 2023. Each cell represents the value for a specific region and year, with darker shades indicating higher values. The color gradient ranges from light (lower values) to deep red (higher values), signifying the increasing trend over time. Reprinted from 'The Global Burden of Periodontal Disease: A Narrative Review on Unveiling Socioeconomic and Health Challenges' by Hashim et al., 2025, licensed under CC-BY 4.0.

The global burden of periodontal diseases, including gingivitis, represents a significant public health challenge with substantial regional disparities (Feng et al., 2025). In recent studies, according to the Global Burden of Disease (GBD), severe periodontitis affects over 1.1 billion people worldwide and ranks as the 11th most prevalent condition. As shown in **Figure 1**, the global burden of periodontal disease has progressively increased from 1990 to 2023, with a marked rise across Africa and Asia, highlighting widening regional disparities and the growing need for targeted preventive strategies (Hashim et al., 2025). It is notable that Africa and Asia show a significant increase in values, especially in the later years, indicating a growing burden in these areas (Hashim et al., 2025).

The transition from gingivitis to periodontitis represents a critical condition in periodontal disease progression. When conditions are left untreated, they can lead to separation of gums from the teeth (Marcenes et al., 2013). Inflammation arising from gingival disease is essential for the breakdown of connective tissue attachments below the cementoenamel junction. This degradation can compromise both the alveolar bone and the surrounding soft tissues that anchor the teeth, resulting in tooth mobility and instability. Without intervention, continued progression of the infectious process may eventually lead to tooth loss (Zhang et al., 2024). The progression of gingivitis to periodontitis leads to a shift in the microflora inside the mouth from the predominance of harmful microbes.

Conventional therapeutic approaches for gingivitis management primarily rely on mechanical plaque removal through scaling, supplemented by chemical plaque control agents. Scaling and root planing is the gold standard for periodontal treatment as the conventional therapy for chronic periodontitis. However, traditional methods have limitations in treating deep pockets, and subgingival area treatment does not eradicate all pathogens because of the complex root anatomy. Chlorhexidine has been suggested as a supplementary treatment alongside scaling and root planning to address the shortcomings of conventional therapies and improve clinical outcomes (Susanto et al., 2022). Chlorhexidine has emerged as the gold standard antiseptic in periodontitis due to its broad antimicrobial spectrum, biocompatibility, low systemic toxic activity in humans and effectiveness in reducing plaque accumulation. Clinical studies show that the addition of chlorhexidine chip therapy to scaling and root planning yields substantial benefits, including greater reductions in probing depth, enhanced clinical attachment gains and improved plaque control compared to scaling and root planning performed independently (Rosa et al., 2021). After four to six weeks of use, chlorhexidine mouth rinse reduced gingivitis by 0.21 on the Gingival Index (0 to 3 scale) compared to placebo, control or no mouth rinse, with a similar effect size found for reducing gingivitis at 6 months (James et al., 2017). But in recent years, the understanding of the microflora inside the mouth in healthy and gingivitis conditions has led the scientific community to develop an interest in the use of natural herbal products rich in the bioactive antimicrobial and bacteriostatic compounds to combat the harmful microflora in the gingivitis conditions. The use of natural products lowers the usage of antibiotics and reduced the chance of development of oral antibiotics resistance microbes.

The focus of this comprehensive narrative review is to explore the role of herbal medicines and natural products used in the treatment of the disease gingivitis. This review discusses the pathogenesis of gingivitis and the role of microbes in it. Consequently, the review discusses the various herbal therapeutic treatments used for gingivitis and the mechanisms involved in them. In addition, the challenges and future perspectives and herbal-based therapeutics for the treatment of gingivitis have been discussed at length.

## 2 Pathophysiological basis of gingivitis

**Figure 2:**
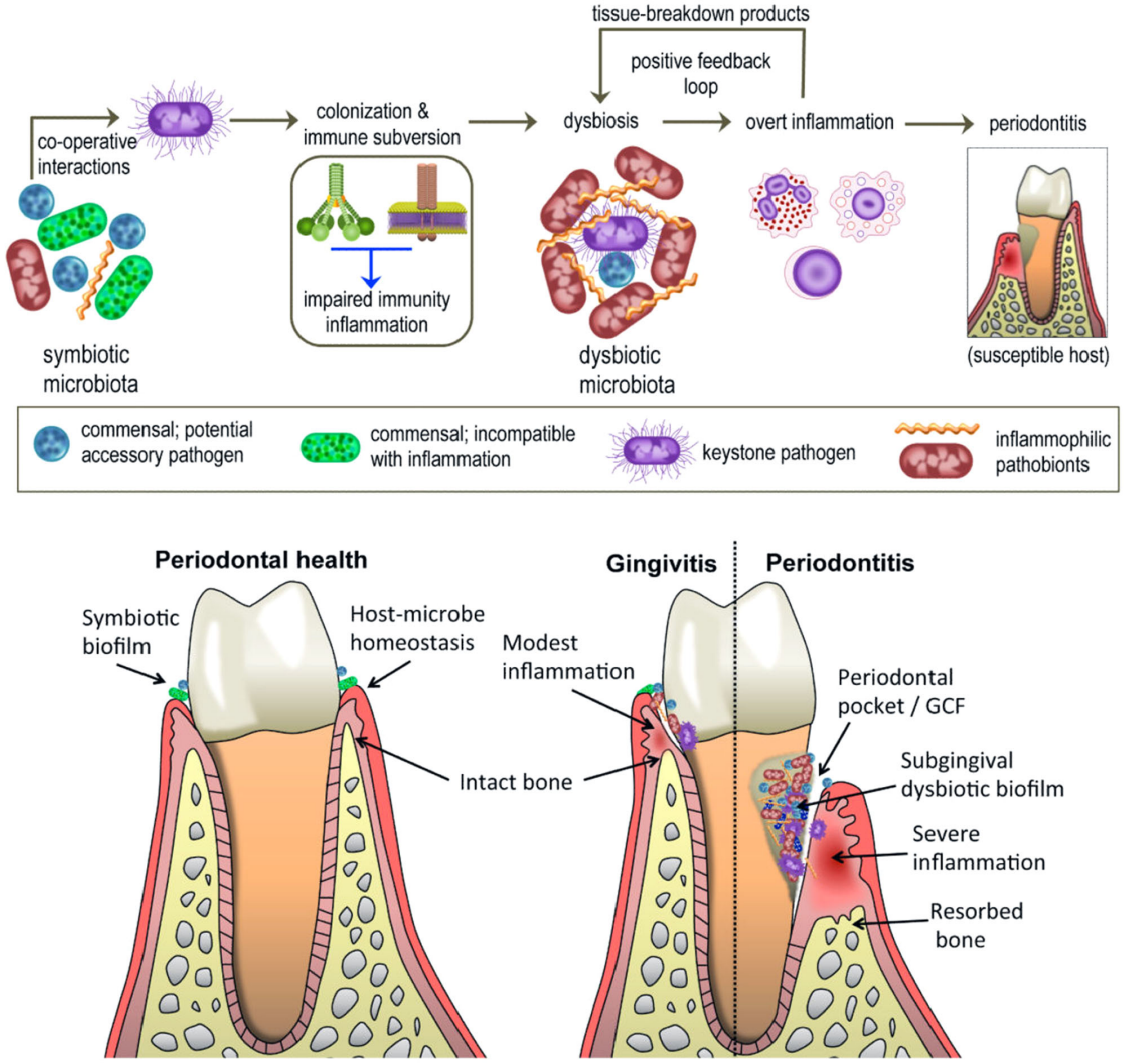
Illustration of how periodontal disease progresses. Healthy gums, with a balanced biofilm and stable host-or microbe environments, also have healthy bone underneath the gums. Gums are inflamed, but no bone loss is seen. There is dysbiosis and an imbalance in the immune system, leading to gum pockets and losses in the alveolar bones during periodontitis stage of progression.

The pathophysiology of gingivitis follows a well-established sequence initiated by the accumulation of dental plaque biofilm at the gingival margin, triggering a cascade of inflammatory responses (**Figure 2**) (Hajishengallis, 2015). Gingival inflammation in response to bacterial plaque accumulation (microbial biofilm) is considered the key risk factor for the onset of periodontitis (Murakami et al., 2018). Activation of innate and adaptive immunity occurs in response to these factors, with subsequent production of pro-inflammatory cytokines. The involvement of cytokines is critical in the pathophysiology of both gingivitis and periodontitis, as they induce activation of immune cells and stromal components. This cellular activation drives local inflammation and causes tissue injury, potentially resulting in the breakdown of periodontal ligaments, gingiva, and supporting alveolar bone (Neurath and Kesting, 2024). However, the pathway from a pre-established gingivitis infection to periodontitis is still far from clear. The lack of keratinization in the active inner epithelial layers of the dental surface is one of the hallmarks of gingivitis. Gingivitis is associated with numerous gram-negative microbial flora known as the red complex. The pathogenic microbes comprising *Treponema denticola*, *Porphyromonas gingivalis*, and *Tannerella forsythia*, adheres to the surface, co-aggregate and infect the buccal epithelial cells (Page and Kornman, 1997; Kinane, 2001). The attack of these pathogens on the host cells is an important virulence way for recurrent infections and evading the defense mechanism of the body (Di Benedetto et al., 2013). The immune response of the body to these pathogens is met with a local inflammatory response and liberation of toxic products. Both acquired and natural immunity is activated with neutrophils penetrating the gingival tissues and expression of antibodies (Kornman, 2008). The breakdown of the supporting structures of the tooth due to the inflammatory molecules and proteases, lead to swollen gums, damaged connective tissues, deepening of periodontal pockets, and loss of the tooth (Trindade et al., 2014).

## 3 Gingivitis and microbial community interaction

Microbial communities present in the subgingival crevices are complex in nature, consisting of Bacteria, Archaea and Eukarya (Dabdoub et al., 2016). The bacterial communities are considered the most diverse and are abundantly found in the subgingival crevices. However, based on the 16s rRNA marker-based findings, it has been suggested that unique microbial signatures exist for each state in healthy, gingival, and periodontal conditions (Diaz et al., 2016). Understanding host-microbial interactions in the oral microbiota is necessary to determine the protective nature of gingival response. The development of gingivitis begins with an increase in the subgingival microbial load that shifts the gingival microbiota towards the Gram-negative and motile bacteria (Lamont et al., 2018). The shift in the microbiota creates new niches suitable for successive waves of species that invade and colonize the subgingival crevices (Narayanan et al., 2023). Further the inflammatory ligands like the Lipopolysaccharide on the Gram-negative bacteria initiate inflammation in the region, which supports the growth of proteolytic and pectinase rich Gram-negative bacteria that uses glycoprotein for energy (Syed and Loesche, 1978; Abusleme et al., 2021). Gingivitis is a well-documented factor in periodontitis. Longitudinal studies on gingivitis associated with gut microbiota that promote periodontitis remain unclear. However, certain evidence from clinical and animal study models has indicated that both microbial load and alterations in the specific community structures are associated with disease progression. Certain keystone species like *P. gingivalis* have been identified to play an important role in microbiota community dysbiosis (Hajishengallis, 2014; Hong et al., 2015; Zhou et al., 2023).

Park and coworkers studied microbial signatures related to health, gingivitis and periodontitis and compared the three states using molecular microbiome analysis (Park et al., 2015). It was found that healthy samples were dominated by *Halomonas hamiltonii*, possibly linked to diet, while periodontitis samples had high levels of *Porphyromonas, Fretibacterium, Rothia*, and *Filifactor*. Gingivitis communities mainly included *Streptococcus, Capnocytophaga, Haemophilus*, and *Leptotrichia.* In the Human Microbiome Project, the healthy state was characterized with the presence of Gram-positive genera like *Rothia, Actinomyces, Streptococcus*, and *Corynebacterium*, with some Gram-negative taxa like *Fusobacterium nucleatum, Veillonella parvula*, and *Capnocytophaga* (Hong et al., 2015). In the periodontitis state, it was characterized by the depletion of Gram-positive bacteria and enrichment of species like *P. gingivalis, Porphyromonas endodontalis, Tannerella forsythia, Treponema* spp.*, Filifactor alocis, Prevotella intermedia, Parvimonas micra*, and *Fretibacterium* spp. Huang et al. studied the gingival microbiome and found the presence of *Actinobacteria* and *Firmicutes* to be associated with health, whereas *Leptotrichia, Prevotella, Fusobacterium*, TM7 *(Saccharibacteria), Porphyromonas, Tannerella, Selenomonas*, uncultured *Lachnospiraceae*, unclassified *Comamonadaceae, Peptococcus, Aggregatibacter, Catonella, Treponema*, SR1 *(Absconditabacteria), Campylobacter, Eubacterium, Peptostreptococcus*, unclassified *Bacteroidaceae, Solobacterium, Johnsonella, Oribacterium*, and unclassified *Veillonellaceae* were enriched during gingivitis (Huang et al., 2014). In the experimental gingival models, it was found that some genera overlap among gingivitis and periodontitis, while in-depth analysis at the species level indicate reveals microbiota enrichment in these conditions differs substantially. This suggests distinct microbial community successions as the disease progresses (Moore et al., 1987; Huang et al., 2014; Diaz et al., 2016; Schincaglia et al., 2017; Abusleme et al., 2021).

**Figure 3:**
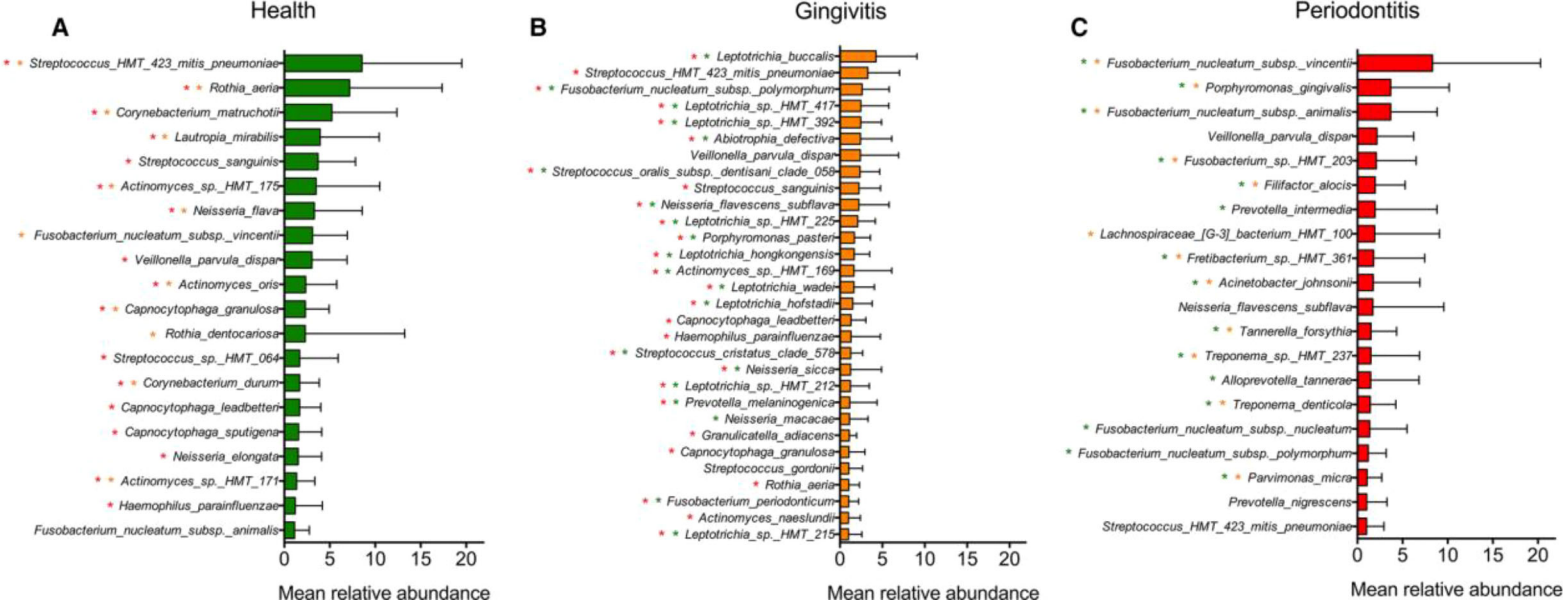
The bacterial community found in healthy **(A)**, gingivitis **(B)**, and periodontitis **(C)** conditions. The green asterisk shows that the species was enriched compared with health; an orange asterisk shows that the species was enriched compared with gingivitis; and a red asterisk shows that the species was enriched compared with periodontitis. Reprinted with permission from 'Microbial signatures of health, gingivitis, and periodontitis' by Abusleme et al., 2021, licensed under © 2021 John Wiley & Sons A/S. Published by John Wiley & Sons Ltd.

Studies on microbial communities and their structure in different states indicate Shannon and inverse Simpson indices, which show higher alpha diversity in the gingival state, whereas comparable diversity between healthy and periodontitis conditions (Abusleme et al., 2013). This suggests that as the condition advances, a subset of the taxa becomes dominant, which reduces evenness among the microbial community despite species richness. The increased microbial species richness and diversity in gingivitis support the idea of an ecological succession model in which plaque accumulation and inflammation provide niches for Gram-negative bacteria without the loss of health associated microbial species (Griffen et al., 2012). In periodontitis, the prevalence of specific proteolytic taxa that becomes numerically dominant for the dysbiotic community could be accounted for reduced diversity with persisting species richness (Abusleme et al., 2013; Camelo‐Castillo et al., 2015; Kirst et al., 2015; Abusleme et al., 2021). The bacteria associated with the healthy, gingivitis, and periodontitis are shown in **Figure 3**.

The pioneer health associated taxa include, *Rothia, Corynebacterium, Lautropia mirabilis, Neisseria, Bergeyella, Kingella oralis*, and facultative anaerobes like *Streptococcus, Actinomyces, Gemella*, and *Capnocytophaga*, which are adapted to oxygenated conditions within shallow and healthy gingival sites (Tanaka et al., 1998). These microbiotas are consistent with a biofilm exposed to oxygen influx and regular mechanical disruption of plaque, thus keeping the communities dominated by health compatible species (Mettraux et al., 1984). The gingival microbial community is associated with low oxygen rich niche that supports the expansion of Gram-negative bacteria and oxygen tolerant bacteria like *Neisseria* and *Streptococcus*. There is a strong enrichment of *Leptotrichia* species, the most abundant gingival species, seen at multiple gingival sites. As the state progresses, the burden of Gram-negative species sharply increases even more than their proportions. *Fusobacterium* shows subspecies-level changes across various stages. *Fusobacterium nucleatum subsp. vincentii* is abundant in health, whereas *F. nucleatum subsp. polymorphum* and *Fusobacterium periodonticum* are enriched in gingivitis (Kistler et al., 2013; Huang et al., 2014; Schincaglia et al., 2017). Periodontitis is marked by a complete shift in the subgingival microbiota towards predominantly Gram-negative and obligate anaerobes. The periodontal condition includes the classical ‘red complex’ and the associated microbes, which indicates the disease progression being marked by periodontal lesions leading to the formation of inflammatory association. With the exception of *F. nucleatum subsp. polymorphum*, all other *F. nucleatum* species are found in similar frequencies in all three states. This supports the idea that *F. nucleatum* serves as a structural support within gingival plaque that help anchor other microbes during the phase transition to periodontitis, where community remodeling occurs (Abusleme et al., 2013; Hong et al., 2015; Abusleme et al., 2021).

All the periodontitis associated species were detected in gingivitis, and most of them were also found in health; however, their frequencies were much lower in health. Species like *Fretibacterium* sp. HMT 361 and *Porphyromonas gingivalis* are among the most frequently detected and abundant taxa in periodontitis, whereas those including *Campylobacter gracilis* and *F. nucleatum subsp. vincentii*, *F. nucleatum subsp. animalis*, and *F. nucleatum subsp. nucleatum*, were common across all three states. These members of the subgingival microbiota expand as the inflammation progresses, positioning gingivitis and periodontitis as points along a succession (Naginyte et al., 2019; Abusleme et al., 2021). The oral cavity is colonized by infectious microbes like protozoans, virus, fungi, and bacteria. Although most of the bacteria in the oral microbiome are harmless, there are certain species of microbial communities that can cause infectious oral diseases in humans like gingivitis and periodontitis (Chinsembu, 2016). These diseases are associated with severe gum inflammation around the tooth and are caused by species such as *Aggregatibacter actinomycetemcomitans*, *Eikenella corrodens*, and *Fusobacterium nucleatum* (Amir Alireza et al., 2014). For a long time, oral cavity maintenance depended on the use of chemical agents, such as fluorides and alcohols, in mouthwashes. Similarly, the use of zinc citrate and triclosan in toothpaste has helped maintain oral hygiene. Synthetic antimicrobials like povidone iodine, phenol derivatives, chlorhexidine, and cetyl pyridinium chloride are also used for the same purpose. In order to prevent bacterial growth, antibiotics such as ampicillin, erythromycin, penicillin, tetracycline, and vancomycin are also frequently used in dentistry (Allaker and Douglas, 2009; Jang et al., 2014). However, it has been found that oral microbial communities show resistance to some chemicals, while some synthetic chemicals are associated with vomiting, diarrhea, and tooth staining. Hence, in the wake of this situation and in light of the need to find cost-effective alternatives to chemical treatments, medicinal plant extracts have been of interest for controlling oral diseases such as gingivitis and periodontitis (Chinsembu, 2016). Plant phytochemicals have been positioned to serve as a bridge between gingivitis and periodontitis and natural herbs. They act on the microbial community and the inflammatory processes that drive periodontitis. Natural herbs rich in polyphenols, flavonoids, and terpenoids exert antimicrobial and biofilm effects against key microbial species. This helps to stabilize the oral microbial community before irreversible tissue damage occurs. Thus, natural herbs provide a rationale for their usage in gingivitis and periodontitis management, offering both host and microbial-specific benefits (Rani et al., 2022; Lin et al., 2025).

## 4 Gingivitis treatment and natural products

The treatment of gingivitis often depends on the decline in the count of pathogenic microbes, which leads to a decrease in inflammation and unhealthy pockets in the mouth. The decrease in pathogenic microbes is achieved by systemic treatment of the mouth with antibiotics. In addition, scaling of the calculus and elimination of plaque are also used for the treatment of gingivitis. But for the recovering of the inflamed tissue, antimicrobial healing is the best method (Steinberg and Friedman, 1988). One of the major hurdles faced with systemic antibiotic treatment is the movement of drugs all over the body, which may lead to many side effects and the development of antimicrobial resistance. In addition, the chemicals commercially used to treat gingivitis can modulate the oral microflora in a negative way leading to staining of teeth, vomiting, and diarrhea. Therefore, plant phytochemical is one of the best alternative and non-conventional medicines that can be used in place of synthetic chemicals and antibiotics. In contrast to the rise in antimicrobial resistance, natural and herbal compounds used in folk medicines can be used for the treatment of gingivitis (Park et al., 2003; Chung et al., 2006; Prabu et al., 2006). As per the estimate of World Health Organization, around 75 percent of the total world population depends on these medicinal plants for healthcare (Park et al., 2003; Chung et al., 2006; Prabu et al., 2006; Ashu Agbor and Naidoo, 2015). These plant phytochemicals are mainly considered antibacterial agents and can cause damage to the walls of both gram-positive and gram-negative bacteria. The antimicrobial performance of these phytochemicals is one of the most valuable treatments for gingivitis. The major lacunae for using plant based products is the lack of data regarding the effect of these phytochemicals from different plants in the treatment of gingivitis healthcare (Anushri et al., 2015). In the next section, various classes of phytochemicals and medicinal plants used for the treatment of oral pathogens have been outlined.

### 4.1 *Camellia sinensis* (green tea)

*Camellia sinensis* or green tea catechins (*Theaceae*) represent one of the extensively studied polyphenolic compounds in oral health research. Epigallocatechin-3-gallate(EGCG), comprising approximately 59% of total catechins in green tea, demonstrates potent antimicrobial and anti-inflammatory properties that make it particularly effective against oral pathogens (Yá et al., 2024). Multiple clinical investigations have confirmed the potent antibacterial activity of epigallocatechin-3-gallate (EGCG) against orally isolated pathogenic bacteria, including *Streptococcus mutans*, *Aggregatibacter actinomycetemcomitans*, *Porphyromonas gingivalis*, and *Prevotella intermedia*. The bactericidal mechanisms operate through compromising bacterial membrane integrity, sequestering critical trace elements such as iron, and disrupting core metabolic pathways. Research conducted in laboratory settings has documented that epigallocatechin-3-gallate (EGCG) can inhibit protein tyrosine phosphatase function. The same compound also substantially diminishes the biofilm-producing capacity of *Fusobacterium nucleatum*, reducing biofilm development by approximately 55.4% compared to control conditions (Kong et al., 2022). The anti-inflammatory effects of green tea catechins are mediated through inhibition of inflammatory cytokines, including IL-17, IL-1β, and TNF-α, as well as modulation of gene expression pathways such as NF-κB. Green tea consumption has increased glutathione-S-transferase activity, thereby reducing oxidative stress and inflammation (Basu et al., 2018).

A multi-arm randomized, double-blinded clinical trial demonstrated that 0.5% green tea mouthwash was as effective as 0.2% chlorhexidine in reducing plaque index, gingival index, and sulcular bleeding index over 21 days. The study showed that green tea mouthwash significantly improved all clinical parameters, with the maximum percentage change found in the chlorhexidine group, followed closely by green tea. Another clinical study involving 30 participants with mild to moderate gingivitis found that green tea mouthwash used twice daily for one week reduced mean gingival index scores from 2.04 ± 0.401 to 0.91 ± 0.364, indicating a shift from moderate to mild inflammation (Lan et al., 2024). Green tea works to protect teeth through several mechanisms. One way is by blocking an enzyme that bacteria used to stick to teeth. When the enzyme is blocked, bacteria can not adhere to tooth surfaces. It also acts by reducing the amount of acid that harmful bacteria produce in the mouth. Research has shown that when people drink green tea every day for a month, their mouth becomes less acidic, the pH goes above 5.5, making the mouth a less hospitable place for cavity-causing bacteria to survive. Additionally, green tea contains compounds that prevent collagenase enzymes from breaking down collagen in tooth and gum tissue. Two catechins in particular, ECG and EGCG, are especially good at blocking collagenase from both human cells and bacteria (Vyas et al., 2021).

### 4.2 *Punica granatum* (pomegranate)

**Figure 4:**
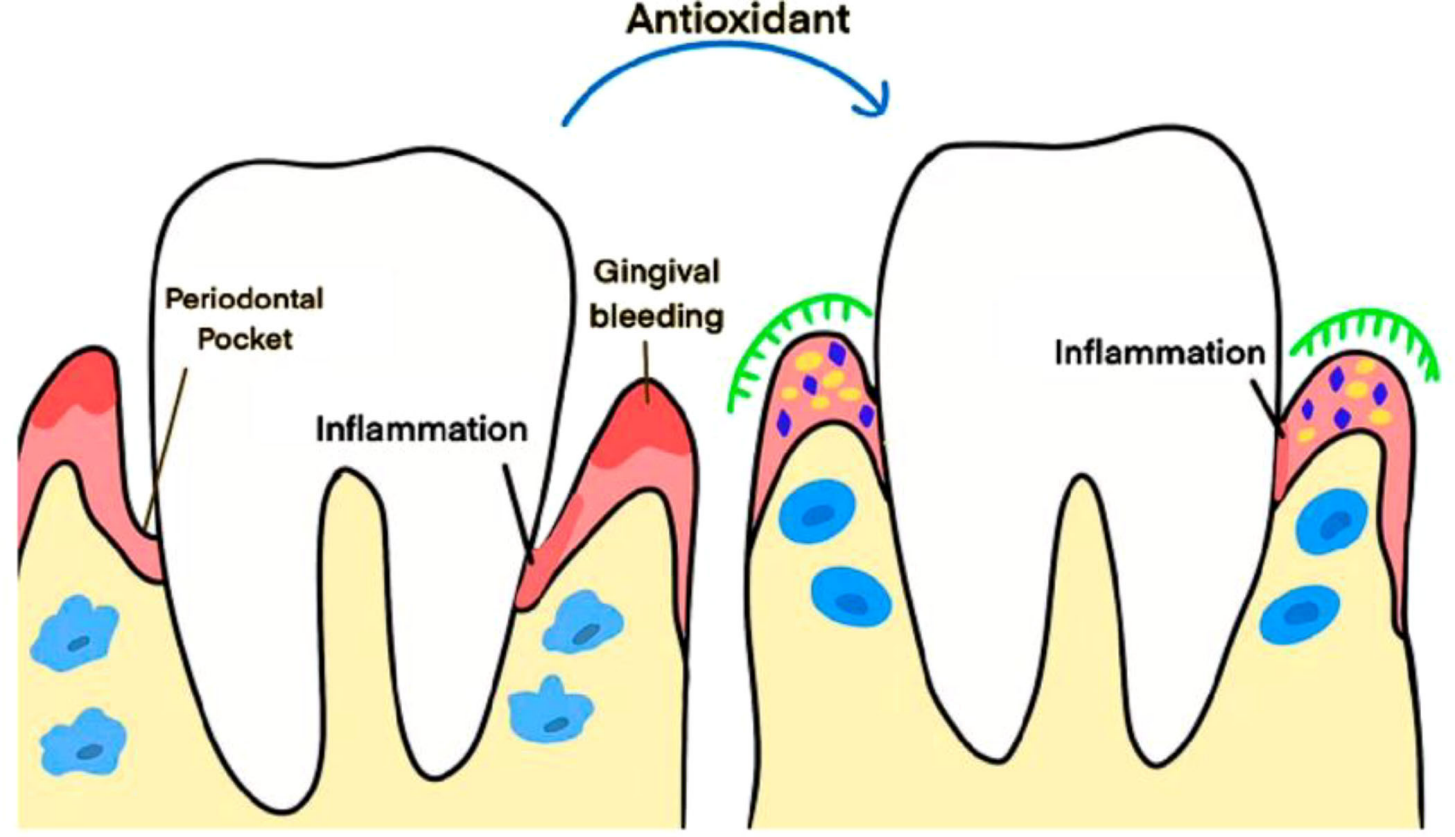
The mechanism of action of antioxidants on periodontal tissue. Reprinted from 'Oxidative stress in periodontitis and the application of antioxidants in treatment: a narrative review' by Liu and Guo, 2025, licensed under CC-BY 4.0.

*Punica granatum* or pomegranate (*Lythraceae*) extracts have gained significant attention in periodontal therapy due to their rich content of punicalagin, ellagic acid, and various flavonoids that demonstrate potent anti-inflammatory and antioxidant properties. Clinical trials consistently show that pomegranate-based interventions can significantly improve gingival health parameters and reduce inflammatory biomarkers. The anti-inflammatory mechanisms of pomegranate compounds involve dose-dependent inhibition of lipopolysaccharides induced nitric oxide and prostaglandin E2 production. Punicalagin and ellagic acid at concentrations of 1, 10, and 50 μM significantly decreased the production of pro-inflammatory cytokines, including TNF-α, IL-1β, and IL-6 in macrophages. The compounds also down-regulate inflammatory mediators’ mRNA expression and suppress phosphorylation of mitogen-activated protein kinases (MAPKs) (Singh et al., 2023). Clinical trials have demonstrated that pomegranate mouthwash improves gingival health with effects comparable to chlorhexidine. Eltay et al. investigation revealed that chronic gingivitis patients experienced decreased plaque index values, gingival index scores, and IL-1β levels following treatment with a pulsatile oral spray preparation formulated with 5% pomegranate extract. The astringent and anti-hemorrhagic properties of pomegranate contribute to reduced gum bleeding comparable to other standard herbal mouthwashes (Eltay et al., 2021; Potra Cicalău et al., 2024). Most of these plant extracts act through the antioxidant phytochemical. The mechanism through which the antioxidants operate on gingivitis is given in **Figure 4**.

### 4.3 *Azadirachta indica* (neem)

*Azadirachta indica* or neem (*Meliaceae*) is recognized as a versatile medicinal plant with potent oral health benefits. The active compounds, including azadirachtin, nimbidin, nimbinin and nimbidol exhibit antibacterial, antifungal, anti-inflammatory, and antipyretic properties. Clinical studies demonstrate that neem extracts can significantly reduce plaque-forming bacteria, including *Streptococcus mutans* and *Lactobacillus* sp*ecies*. In a double blind randomized controlled trial, researchers combined 0.19% neem treatment with standard mechanical cleaning to manage plaque-induced gingivitis (Gawish et al., 2023). A randomized clinical trial comparing neem mouthwash to *Camellia sinensis* and chlorohexidine showed that both herbal extracts improved the gingival index, plaque index, oral hygiene index, and pH levels better than chlorohexidine, with green tea slightly outperforming neem in plaque reduction. Another study demonstrated that neem and chlorohexidine mouthwashes showed similar efficiency in reducing plaque index, sulcular bleeding index, and gingival index after 4 weeks of use (Safiaghdam et al., 2018). The mechanisms of neem effectiveness include inhibition of glucosyltransferase activity, reduction of bacterial adherence to tooth surfaces, and anti-inflammatory effects through prostaglandin inhibition. Neem also demonstrates inhibitory effects on matrix metalloproteinase-2 and MMP-9, both associated with extracellular matrix breakdown in periodontitis. Regular use of neem-based oral care products can decrease plaque formation, prevent cavities, and strengthen immune system function (Gawish et al., 2023).

### 4.4 *Aloe vera (L.) Burm.f.* (aloe vera)

Aloe vera *[Asphodelaceae (Liliaceae)]* demonstrates significant potential in periodontal therapy through its anti-inflammatory, antimicrobial, and tissue-healing properties. Clinical trials show that aloe vera gel and rinses can effectively reduce the gingival index, sulcular bleeding index, and bleeding on probing. A double-blind study involving 45 subjects found that daily rinsing with 15ml of aloe vera solution significantly decreased gingival and bleeding indices after three months, with enhanced effects when combined with scaling and root planing (Safiaghdam et al., 2018). The bioactive components of aloe vera, including polysaccharides, anthraquinones and glycoproteins, contribute to its therapeutic effects such as antiviral, anti-cancer, and anti-ulcer effects. Aloe vera demonstrates protective effects on periodontitis ligament cell viability and can reduce neutrophil infiltration in aggressive periodontitis models (Rezaei-Tazangi et al., 2024). Aloe vera acts as an antiseptic and controls inflammation, and decreases gum bleeding. It also harbors antifungal activities and contains carboxypeptidase, which reduces pain by the deactivation of bradykinin (Keisuke et al., 1976; Tello et al., 1998; Bautista-Pérez et al., 2004). Abdelmonem et al. showed that the addition of *Aloe vera* along with scaling and root planning enhances the gingivitis treatment (Abdelmonem et al., 2014).

### 4.5 *Curcuma longa L.* (turmeric)

Turmeric, (*Zingiberaceae*) obtained from the rhizomes of the *Curcuma longa L.* plant is an orange-yellowish spice with curcumin as the main constituent. Curcumin reduces inflammation by lowering dopamine levels, thereby used to treat swellings (Ammon et al., 1993; Nagpal and Sood, 2013). Turmeric water can be used as a mouthwash to reduce pain and inflammation. Turmeric can be ground and applied to painful teeth sites to reduce swelling. Gingivitis and periodontitis can be treated by applying turmeric, salt, and mustard oil on the teeth and gums that act on the subgingival crevices to reduce inflammation. Turmeric is considered having anti-cariogenic activity, as the essential oil isolated from it inhibits proliferation and acid generation of Streptococcus mutants up to 4 mg/ml (Lee et al., 2011). Mouthwash consisting of turmeric can be used for the management of conventional plaque. ‘Curenext’ is a topical gel used to treat gingivitis and periodontitis in which 10mg/g of *Curcuma longa* L. extract is used (Sharma and Kalsi, 2016). To get relief from periodontitis, 1% curcumin is often used for subgingival irrigation that reduces redness, pain, and inflammation (Monisha and Ramamurthy, 2019). It reduces inflammation and swelling of the connective tissue by specifically inhibiting the inflammatory mediators, which provide a better solution for the inflammatory response than saline and chlorhexidine irrigation. It enhances wound healing by changing the growth factor transcription (Cikrikci et al., 2008; Mazzarino et al., 2014).

### 4.6 *Ocimum sanctum L.* (tulsi)

The chemical composition of *Ocimum sanctum L. (Lamiaceae)* is complex, and its active antibacterial characteristics lie in its essential oils. The main components included in the essential oils are caryophyllene oxide, caryophyllene, clemene, eugenol and germacrene-A, which contributes to its antibacterial properties, thus making it effective against both Gram-positive and Gram-negative bacteria (Mondal et al., 2009; Gupta et al., 2014). In a study by Gupta and coworkers, it was found that the effectiveness of Ocimum sanctum like mouthwash could be used which to treat gingivitis and periodontitis like Chlorhexidine (Gupta et al., 2014). In a study by Hosadurga, it was found that 2% tulsi gel could be potentially used for the treatment and management of gingivitis (Hosadurga et al., 2015). Tulsi leaf extract boosts interleukin-4, T-helper cells, and natural killer cells, enhancing host immune responses to infections. This supports its adjunctive role in periodontal therapy alongside conventional treatments (Mondal et al., 2011; Pai et al., 2019).

### 4.7 *Mentha×Piperita L.* (peppermint)

Peppermint belonging to the Lamiaceae family, is known for its analgesic, anti-inflammatory, and antibacterial effects. This is owed to its phenolic and essential oil constituents that help in providing relief from dental pain, swelling, inflammation, and gingival conditions (Kumar et al., 2009). The chemical constituents of peppermint include menthol, menthone, and flavonoids that deliver cytoprotective, antioxidant, and antiulcer activities, effectively targeting carcinogenic and oral pathogens. 4–63g daily leaf capsules or tablets are used as a mouthwash for post-periodontitis healing, or it is incorporated into gels/rinses for bacterial controls (Mimica-Dukic and Bozin, 2008; Elgendy et al., 2013).

### 4.8 *Trifolium pratense L.* (red clover)

Red clover *(Fabaceae*) is known to show anti-inflammatory activity against gingivitis and periodontitis owing to its polyphenolic composition. The mouthwash prepared from red clove can be used to irrigate gingival crevices, while the gel isolated from leaves and flowers can be applied on the sites possessing antibacterial activities (Ramos et al., 2012; Kumar et al., 2013).

### 4.9 *Matricaria chamomilla L.* (chamomile)

Chalomile *(Asteraceae)* is one of the highly preferred traditional medicines for the treatment of gingivitis and gum associated problems. This is attributed to its flavonoids, sesquiterpenes, polyacetylenes, and coumarins, which reduce swelling and inflammation. This occurs due to its role in modulating the microbial community, thereby making it ideal candidate for mouthwash compared to Chlorhexidine (Srivastava et al., 2009; Singh et al., 2011). Its active compounds like apigenin and chamazulene, inhibit inflammation and microbial growth in oral tissues, supporting its use in capsules, tablets, tinctures, lotions. Lucena observed gingivitis bleeding was significantly reduced upon the use of Chamomile mouthwash, while combination of chamomile and pomegranate mouthwash, detailed by Batista and coworkers (de Lucena et al., 2009; Batista et al., 2014). This was comparable to 0.12% chlorhexidine mouthwash and parallels were drawn on the basis on its anti-inflammatory action. It has also been established by Agarwal et al. that chronic periodontitis could also be managed by using chamomile (Taheri et al., 2011; Agarwal and Chaudhary, 2020).

### 4.10 *Calendula officinalis* or Mary’s gold (Asteraceae) extract

represents anti-inflammatory, antimicrobial, and wound healing properties that are beneficial for gingivitis management. The therapeutic compounds in calendula include flavonoids, particularly quercetin, saponins, and essential oils that contribute to its healing properties. The wound-healing properties of calendula are mediated through stimulation of epithelial cell proliferation, enhanced angiogenesis, and increased collagen synthesis. Clinical studies have shown that calendula oral rinse can improve inflammation, plaque index, and bleeding in gingivitis patients. The gentle nature of calendula preparations makes them suitable for patients’ sensitive oral tissues (Molayem and Pontes, 2023). *Rosemary officinalis* or Rosemary (*Lamiaceae)*has demonstrated significant antimicrobial and anti-inflammatory properties relevant to oral health. The active compounds in rosemary include rosemaric acid, carnosic acid, and various essential oils that contribute to its therapeutic effects. A randomized controlled trial examining rosemary-based toothpaste found an effective treatment of gingival bleeding and bacterial plaque reduction compared to conventional toothpaste. The study showed that rosemary preparations provided similar clinical benefits to standard treatments while offering the advantage of natural origin. However, the study also noted that some individuals may experience allergic reactions to rosemary, requiring caution in sensitive patients (Valones et al., 2019).

### 4.11 Other medicinal plants

*Salvia officinalis* L. or Sage mouth rinse has antiseptic and astringent properties and helps in treating gingivitis (Narayanan and Thangavelu, 2015). The extracts and essential oils from Sage exhibits antibacterial, antifungal, and anti-inflammatory effects. In traditional medicine, the filtrate from sage leaves is used for gargling, which leads to significant improvement in the gingivitis condition. *Zingiber officinale* or ginger (*Zingiberaceae*) has demonstrated antimicrobial activity against oral pathogens and shows promise in reducing periodontal inflammation. Research on herbal nano formulation-assisted mouth paint prepared using lemongrass and ginger mediated titanium dioxide nanoparticle showed antimicrobial activity against oral pathogens, including *Candida albicans*, *Staphylococcus aureus*, *Streptococcus mutans*, and *Enterococcus faecalis* (Rifaath et al., 2023).

### 4.12 Comparative analysis

The above section discussed shows a comparison of antimicrobial efficacy, anti-inflammatory activity, biofilm inhibition and tissue healing potential of natural products in gingivitis management. *Camellia sinensis* (green tea), exhibit a strong antimicrobial and antibiofilm activity against major periodontal pathogens including *Streptococcus mutans*, *Aggregatibacter actinomycetemcomitans, Porphyromonas gingivalis*, *and Fusobacterium nucleatum.* Its catechin EGCG produces clinical results similar to chlorohexidine by combining bacterial action, inhibition of bacterial adhesion suppression of collagens activity and modulation of inflammatory cytokines. On the other hand, *Azadirachta indica* (neem) shows strong robust plaque reducing effects through inhibition of bacterial adherence and glucosyltransferase activity, while clinical trials indicates similar efficacy to chlorohexidine, slightly less effective than green tea. *Punica granatum* (pomegranate) and *Curcuma longa L.* (turmeric)have relatively stronger anti-inflammatory effects than green tea and neem that mainly target microbial burden. Pomegranate polyphenol reduces MAPK signaling and pro-inflammatory cytokines results reduction in gingival inflammation and bleeding. Curcumin exhibits relatively weaker anti-microbial action, frequently outperforms saline in reducing redness, pain, and swelling, and shows similar effects to chlorohexidine in subgingival irrigation. On the other hand, *Calendula officinalis* (Mary’s gold), when used in conjugation with scaling and root planning, exhibits moderate antimicrobial activity but excels in promoting gingival tissue healing, epithelial regeneration, and reduction of bleeding. *Ocimum sanctum L*.(Tulsi), *Matricaria chamomilla L.* (chamomile) and essential oil rich plants such as *Mentha×Piperita L.* (peppermint), *Salvia officinalis L.* (sage), *Zingiber officinale* (ginger), and *Rosemary officinalis* (rosemary) that provides supportive antibacterial, anti-inflammatory and immunomodulatory effects. In the treatment of gingivitis, chamomile and tulsi have shown similar clinical outcomes compared to chlorohexidine. Overall, this comparative analysis shows that natural products have different clinical strengths and dominant mechanisms of action, which supports their selective use rather than a consistent therapeutic replacement in gingivitis treatment (**Table 1**).

**Table 1:** Medicinal plants and part of the plant used for the clinical trials.

Name of the plant	Part of the plant used	Form/extract studied	Reference
Green tea	Leaves (catechins)	Mouthwash/dentifrice/local delivery	(Basu et al., 2018),
Pomegranate	Fruit peel/extract	Extract mouthwash/gel	(Singh et al., 2023)
Neem	Leaves	Extract mouthwash/gel	(Chatterjee et al., 2011)
Aloe vera	Leaf gel	Gel, mouthwash	(Safiaghdam et al., 2018)
Turmeric	Rhizome	Curcumin-rich extracts	(Sharma and Kalsi, 2016)
Tulsi	Leaves	Mouthwash	(Mondal et al., 2011)
Peppermint	Leaves/oil	Included in mouth rinses	(Madhavan and Sreedevi, 2018)
Red clover	Aerial parts	Limited clinical periodontal data	(Ramos et al., 2011)
Chamomile	Flowers	Mouth rinse/gel	(Valmy et al., 2025)
Marigold	Flowers	Polyherbal mouthwash	(Shrinidhi Maji Shankar et al., 2017)
Rosemary	Leaves/oil	Polyherbal mouthwash	(Bozin et al., 2007)

## 5 Challenges and future perspectives

One of the most significant challenges in herbal interventions for gingivitis is the lack of standardized formulations and dosing protocols. Variability in herbal formulations affects reproducibility and clinical efficacy due to lack of standardization in concentration, purity, and preparation methods (Lan et al., 2024). The absence of a standardized quality control profile poses a significant barrier to its widespread acceptance. Different studies use varying dosages, formulations like gels, mouth washes and pastes, and treatment durations, leading to inconsistencies in reported outcomes (Beattie, 2024). The gut microbiome exhibits substantial inter-individual variation that may influence the effectiveness in dietary interventions. Diet, genetics and the gut microbiome could explain 9.3%, 3.3% and 12.8% of inter-individual variations in the whole plasma metabolome. Microbial variation in both gut and oral environments is predominantly determined by individual-specific factors, responsible for roughly 70–88% of taxonomic differences, whereas ethnic background accounts for a more limited 2-10%. These intrinsic factors substantially overshadow any changes produced by brief dietary modifications. This substantial individual variation makes it difficult to predict gut microbiota and host responses to herbal interventions (Chen et al., 2022).

Recent advances in high-throughout sequencing and computational biology have fundamentally transformed how we understand the oral microbiome and its connection to periodontal disease. By combining data from multiple oral microbiome studies, researchers have been able to identify consistent microbial signatures that distinguish healthy gums from periodontitis. This work has pinpointed 54 bacterial species and 26 metabolic markers that show reliable differences between diseased and healthy states, with these patterns holding true across diverse populations spanning five different countries (Geng et al., 2024). New investigations reveal that when machine learning approaches are applied to analyze the taxonomic composition of oral microbiomes, they can accurately identify individuals suffering from periodontitis versus healthy individuals. The diagnostic performance is notably strong, correctly identifying 93% of diseased cases (sensitivity) while maintaining 84% accuracy in recognizing healthy controls (specificity). This work demonstrates the considerable promise of integrating microbial profiling with sophisticated computational analysis for periodontal disease detection (Soueidan et al., 2024). Technology powered by artificial intelligence is now playing a meaningful role in creating personalized herbal approaches for maintaining oral health. By applying machine learning and deep learning techniques, scientists can now shift through large volumes of health and dietary information to identify patterns and draw clinically relevant conclusions. When researchers examined eleven separate studies that tested AI-guided dietary/herbal intervention programs, they found that people following these recommendations experienced benefits across multiple areas: their blood sugar remained more stable, their overall metabolic function improved, and their psychological well-being enhanced. The data showed a notable 39% reduction in how severely participants experienced symptoms, alongside better disease management overall (Wang et al., 2025).

The field of oral health treatment is witnessing the emergence of next-generation polyherbal and multi component nutraceutical combinations as a promising new therapeutic option. Unlike their conventional counterparts, next-generation probiotics/herbal formulations are designed to deliver targeted therapeutic benefits through microbiome modification, performing better than conventional probiotics and providing new treatment options. These advanced formulations differ from traditional probiotics by utilizing more refined biological mechanisms and being specifically created to manage particular health conditions through precise intervention at critical microbial pathways (Jan et al., 2024). Scientists Varoni and Iriti (2016) have proposed the term ‘odontonutraceuticals’ to describe plant chemicals that can help prevent and treat oral diseases. When herbal products are combined in formulations together, they show powerful antimicrobial effects that kill harmful bacteria, reduce inflammation in gum tissues and provide antioxidant protection to prevent tissue damage. These beneficial effects come from a complex mixture of plant compounds, including flavonoids, terpenoids, alkaloids, and phenolic substances that work together (Anwar et al., 2025)

## 6 Conclusion

Gingivitis is one of the most widespread and common oral diseases worldwide. The modulation of the oral microflora and the development of antimicrobial resistance due to the chemicals and antibiotics used for the management of gingivitis have demonstrated that herbal and traditional medicine are a safer and effective approach for treating gingivitis. Phytochemicals from medicinal plants such as green tea, pomegranate, neem, aloe vera, and sage exhibit strong antimicrobial and anti-inflammatory activities, and are comparable to conventional agents like chlorhexidine with fewer adverse effects. But the clinical translation of these traditional medicines faces several challenges. Some of the major challenges are lack of formulation standardization, inconsistent dosing strategies, variability in bioactive compound concentrations, and significant inter-individual differences in oral microbiome composition. In conclusion, the advancement of science and solving the current lacunae and drawbacks in the future can lead to odontonutraceuticals and advanced polyherbal formulations for the treatment and management of gingivitis.
